# The exported *Plasmodium berghei* protein IBIS1 delineates membranous structures in infected red blood cells

**DOI:** 10.1111/j.1365-2958.2012.08004.x

**Published:** 2012-02-21

**Authors:** Alyssa Ingmundson, Carolin Nahar, Volker Brinkmann, Maik J Lehmann, Kai Matuschewski

**Affiliations:** 1 Max Planck Institute for Infection BiologyCharitéplatz 1, 10117 Berlin, Germany; 2 Department of Molecular Parasitology, Humboldt UniversityPhilippstrasse 13, 10117 Berlin, Germany

## Abstract

The importance of pathogen-induced host cell remodelling has been well established for red blood cell infection by the human malaria parasite *Plasmodium falciparum*. Exported parasite-encoded proteins, which often possess a signature motif, termed *Plasmodium* export element (PEXEL) or host-targeting (HT) signal, are critical for the extensive red blood cell modifications. To what extent remodelling of erythrocyte membranes also occurs in non-primate hosts and whether it is in fact a hallmark of all mammalian *Plasmodium* parasites remains elusive. Here we characterize a novel *Plasmodium berghei* PEXEL/HT-containing protein, which we term IBIS1. Temporal expression and spatial localization determined by fluorescent tagging revealed the presence of IBIS1 at the parasite/host interface during both liver and blood stages of infection. Targeted deletion of the IBIS1 protein revealed a mild impairment of intra-erythrocytic growth indicating a role for these structures in the rapid expansion of the parasite population in the blood *in vivo*. In red blood cells, the protein localizes to dynamic, punctate structures external to the parasite. Biochemical and microscopic data revealed that these intra-erythrocytic *P. berghei*-induced structures (IBIS) are membranous indicating that *P. berghei*, like *P. falciparum*, creates an intracellular membranous network in infected red blood cells.

## Introduction

Malaria is an arthropod-borne infectious disease caused by obligate intracellular eukaryotic pathogens of the genus *Plasmodium*. Within their mammalian hosts, *Plasmodium* parasites must adapt to the intracellular environment of two vastly different host cell types for their development. Initially, infectious sporozoites invade and develop within hepatocytes in the liver. During this clinically silent phase, parasites develop and efficiently replicate into thousands of merozoites capable of infecting red blood cells (Silvie *et al*., 2008a). Once released from the liver, these merozoites infect red blood cells, a unique cellular niche, which is devoid of any organelles and lacks vesicular transport. This blood stage of infection is responsible for the devastating disease symptoms of malaria (Haldar *et al*., 2007).

*Plasmodium falciparum*, the species causing the most severe disease in humans, is known to drastically remodel the red blood cells in which they develop. Infection leads to deformation of the red blood cell surface, and to the generation of multiple compartments in the red blood cell cytoplasm, the most prominent of which are membranous structures termed Maurer's clefts (Maurer, 1902; Trager *et al*., 1966; Elmendorf and Haldar, 1994; Lanzer *et al*., 2006; Haeggstrom *et al*., 2007; Hanssen *et al*., 2010). These modifications of the host cell architecture are important to change the adherence properties of the infected red blood cell, to avoid splenic clearance, and to alter the red blood cell permeability to allow the acquisition of nutrients (Ginsburg *et al*., 1983; Rowe *et al*., 2009). Additional structures, including dots containing J-domain-containing HSP40s termed J-dots (Külzer *et al*., 2010), cylindrical structures, which likely tether Maurer's clefts (Pachlatko *et al*., 2010), and additional membrane vesicle populations (Hanssen *et al*., 2010) have recently been identified, by fluorescent labelling of novel exported *P. falciparum* proteins and high resolution microscopy of infected erythrocytes. While experimental genetics has shown that deletion of some Maurer's-cleft-localized proteins affects the surface localization of *P. falciparum* virulence antigens, i.e. *Pf*EMP1 (erythrocyte membrane protein 1) and KAHRP (knob-associated histidine-rich protein (Cooke *et al*., 2006; Dixon *et al*., 2011), due to the lack of *P. falciparum in vivo* models the precise and distinct functions of these compartments in the context of a parasite infection and in malaria disease progression remains elusive.

Interestingly, such dramatic red blood cell modifications have not been yet been described for non-primate *Plasmodium* species, including the rodent parasite *P. berghei*, raising the possibility that extensive remodelling of host erythrocytes was acquired later in evolution after the diversification of the superorder *Euarchontoglires*. Alternatively, similar parasite-induced structures might have been missed thus far. In favour of the latter hypothesis, *P. berghei-*infected erythrocytes also display altered adherence properties (Franke-Fayard *et al*., 2005) and the intracellular parasites presumably also need to acquire nutrients.

Because the pre-erythrocytic stages of *Plasmodium* infection occur in hepatocytes with intact typical cellular architecture, intracellular parasites face different challenges in these cells, and it is unclear if parasites developing in the liver must remodel their host cells to the same extent as *P. falciparum* remodels red blood cells. Nevertheless, CSP (circumsporozoite protein) has been shown to reprogram signalling pathways in infected hepatocytes (Singh *et al*., 2007), and host cholesterol and lipid binding proteins are important for the development of the liver stage *Plasmodium* parasites (Bano *et al*., 2007; Rodrigues *et al*., 2008). This evidence of host parasite interaction during liver infection stages prompted us to search for additional parasite proteins that may mediate such interactions.

In human red blood cells, much of the host cell rearrangement is mediated by parasite proteins exported to the host cell. Many of these proteins possess an N-terminal amino acid sequence known as a PEXEL (*Plasmodium* export element) or HT (host-targeting) motif consisting of a canonical signal sequence followed by R/KxLxE/Q/D, which signals the export of these proteins across the parasitophorous vacuole containing the parasite into the host erythrocyte (Hiller *et al*., 2004; Marti *et al*., 2004). In this study, we systematically searched for *Plasmodium* proteins that possess predicted PEXEL/HT motifs that are expressed in liver stages of infection with the hypothesis that these proteins may be modulating host cell processes during liver infection stages. We focused on one protein, encoded by *PBANKA_136550*, that is shared between liver and blood infection stages. In blood stages of infection the protein localizes to discrete, previously undescribed structures in the host cell cytoplasm. We have termed these novel structures intra-erythrocytic *P. berghei*-induced structures (IBIS).

## Results

### Identification of IBIS1 as an exported liver stage protein

The *P. yoellii* orthologue of *PBANKA_136550*, *PY05401*, was previously demonstrated by microarray analysis to be actively transcribed in liver stage parasites (Tarun *et al*., 2008). In addition, both PBANKA_136550 and PY05401 possess PEXEL/HT motifs as predicted by the described ExportPred algorithm (Sargeant *et al*., 2006). This protein is apparently restricted to rodent malaria parasite species (Fig. S1), and herein, we propose to call PBANKA_136550 IBIS1 (Intra-erythrocytic *P. berghei*-Induced Structures protein 1).

**Fig 1:**
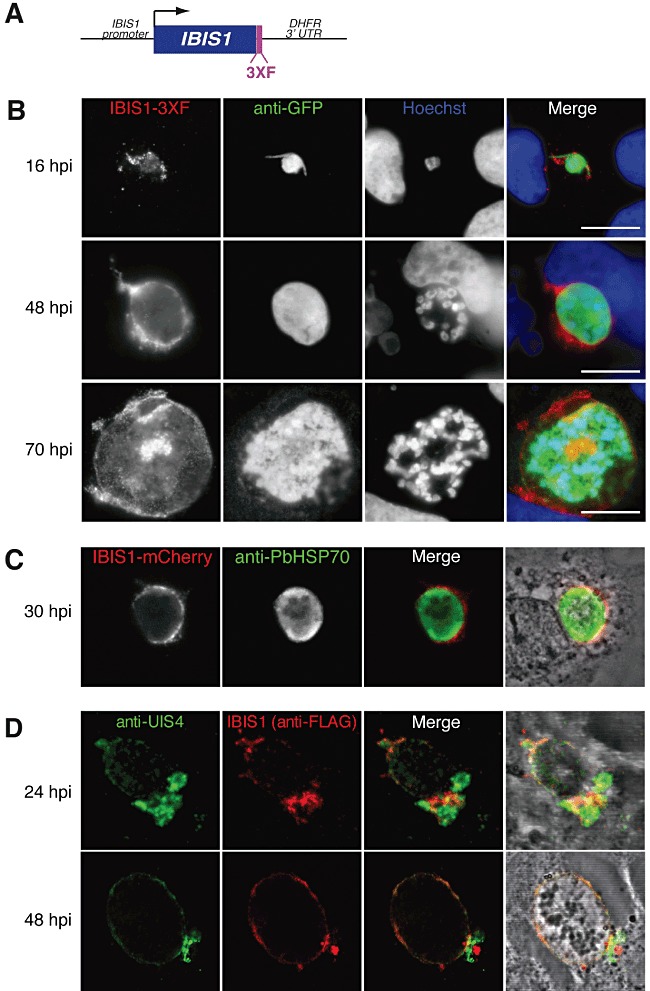
IBIS1 is secreted from liver stage parasites and localizes to the PVM in domains distinct from UIS4. A. Schematic of the transgenic triple FLAG-tagged IBIS1 parasites (*IBIS1-3xF*). The recombinant parasite line contains a carboxy-terminal fusion of three copies of the octapeptide FLAG tag (DYKDDDDK) obtained by insertional tagging of the endogenous *IBIS1* gene (see Fig. S2 for details). B. Detection of the IBIS1-3xF fusion protein by epifluorescence microscopy. Hepatoma cells were infected with *IBIS1-3xF* sporozoites and fixed at the indicated time points post infection. Samples were stained with antibodies against FLAG (left) and GFP (centre left) in order to visualize IBIS1 and the parasites respectively. Nuclei were stained with Hoechst (centre right). Merged pictures (right) are colour-coded. Note that the IBIS1 signal extends into the host cell cytoplasm at all time points during intra-hepatic development. C. IBIS1 tagged with mCherry has a similar localization during liver infection as IBIS1-3xF. Hepatoma cells were infected with *IBIS1-mCherry* parasites and fixed 30 h post infection. Parasites were visualized with an antibody against *Pb*HSP70 and imaged with epifluorescence microscopy. Pictures merged with the phase-contrast images (right) are colour-coded. D. IBIS1 and UIS4 label distinct subdomains of the parasitophorous vacuolar membrane. Hepatoma cells infected with *IBIS1-3xF* parasites were fixed 24 or 48 h post infection, and IBIS1 and UIS4 were detected with the indicated antibodies. Shown are maximal projections of confocal z-series. Pictures merged with the phase-contrast images are shown on the right. Scale bars, 5 µm.

To analyse protein expression and localization of IBIS1 during liver stages of infection, we generated transgenic *P. berghei* ANKA lines expressing IBIS1 tagged with either the fluorescent protein mCherry or a triple FLAG epitope tag (3xF) from the endogenous *IBIS1* promoter (Fig. S2). Using a single cross-over integration strategy we replaced the endogenous copy with the tagged versions, permitting a physiological assessment of the tagged proteins. This tagging was performed in both *P. berghei* ANKA and in a *P. berghei* ANKA line constitutively expressing GFP (Janse *et al*., 2006). Tagged protein of the predicted size was detected by Western blot analysis in both the *IBIS1-mCherry* and *IBIS1-3xF* parasite lines (Figs 5 and S3). A complementary attempt to raise specific peptide antibodies was unsuccessful, as judged by non-specific signals in the absence of IBIS1 (data not shown). Beginning 16 h after infection of hepatoma cells with transgenic *IBIS1-3xF P. berghei* parasites, we were able to detect IBIS1 surrounding the parasite in a pattern indicative of the parasitophorous vacuole membrane (PVM) and to the extended tubular vesicular network (TVN) surrounding the PVM (Fig. 1B) (Mueller *et al*., 2005). The intensity of the IBIS1-3xF signal increased over time, and a similar localization pattern extending into the host cell cytoplasm was consistently observed also at late time points of infection, when liver stages mature to large schizonts. Imaging of hepatoma cells infected with *IBIS1-mCherry* parasites showed localization patterns similar to those of *IBIS1-3xF* (Fig. 1C). However, we detected more structures extending into the hepatoma host cells with the *IBIS1-3xF* parasites, presumably because of the sensitivity of the anti-FLAG antibody in immunofluorescence relative to the mCherry signal after fixation. When compared with the localization pattern of the described PVM-resident protein UIS4 (Mueller *et al*., 2005), a liver stage-specific member of the early transcribed membrane proteins (ETRAMPs) (Spielmann *et al*., 2003), IBIS1 appears to delineate the same compartment. We therefore used an anti-UIS4 antiserum and co-stained fixed *IBIS1-3xF*-infected hepatoma cells with monoclonal anti-FLAG antibody (Fig. 1D). Intriguingly, the signals from the two proteins do not completely overlap, indicating that these proteins localize to distinct domains on the PVM. This observation is consistent over the course of liver stage infection in which these two proteins are simultaneously expressed.

We conclude that in *P. berghei* liver infection, the PEXEL/HT-containing IBIS1 protein remains detectable on the PVM throughout liver stage development and localizes to subdomains distinct from the PEXEL/HT-negative UIS4 protein.

### The contribution of IBIS1 to parasite development

**Fig 2:**
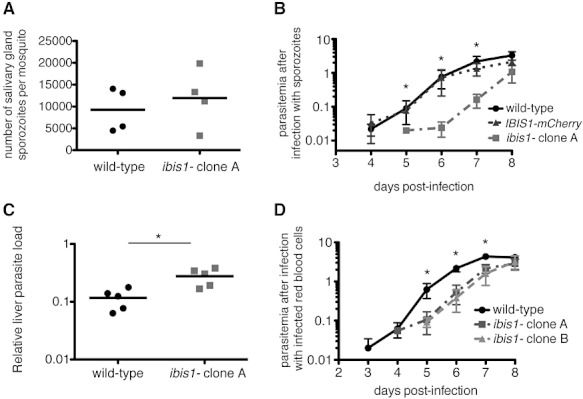
IBIS1 contributes to efficient growth of *P. berghei* in red blood cells *in vivo*. A. *ibis1*^-^ parasites develop normally in mosquitoes. Mosquitoes were infected with wild-type or *ibis1*^-^ parasites in parallel and salivary gland sporozoites were dissected and quantified at least 17 days post infection. Individual data points represent the average number of sporozoites per mosquito from independent feedings from which at least 20 mosquitoes were dissected. The differences between wild-type and *ibis1*^-^ are not significant: *P* > 0.1 by paired two-tailed Student's *t*-test. B. *ibis1*^-^ parasites have delayed growth in mice after sporozoite infection. The parasitemia (% of peripheral red blood cells infected) was assessed after IV injection of either *ibis1^-^* or wild-type sporozoites into mice (1000 sporozoites per mouse). *P < 0.05 by unpaired two-tailed Student's *t*-test (*n* = 6). C. Normal liver stage maturation of *ibis1*^-^ parasites *in vivo*. Forty hours after intravenous injection of either *ibis1^-^* or wild-type sporozoites, relative parasite burden in the liver was measured by qPCR analysis of extracted RNA (*n* = 5). Levels of *Pb18s* rRNA were normalized to levels of mouse *GAPDH* RNA. *P < 0.05 by unpaired two-tailed Student's *t*-test. D. Delayed *in vivo* blood stage development in mice infected with *ibis1*^-^ parasites. Blood parasitemia of mice was measured following intravenous injection of 1000 red blood cells infected with either *ibis1^-^* clone A, *ibis1*^-^ clone B or wild-type parasites. *P < 0.05 by unpaired two-tailed Student's *t*- test comparing each *ibis1*^-^ clone independently to wild-type parasites (*n* = 5).

To analyse the influence of *IBIS1* on parasite growth and development, an *ibis1* knockout line was generated in *P. berghei* ANKA by replacing the *IBIS1* gene via homologous recombination with *Tgdhfr/ts* (Fig. S4). After *in vivo* cloning by limited dilution, *ibis1*^-^ parasites were analysed for their capacity to complete all stages of the life cycle in both mosquitoes and mice (Table S2). The *ibis1*^-^ parasites exhibited no impairment in gametocyte production when mixed blood stages from infected mice were assessed (Fig. S5A). Furthermore, after feeding to *Anopheles stephensi* mosquitoes there was no detectable reduction in salivary gland-associated sporozoites (Fig. 2A). When sporozoites were injected into susceptible C57Bl/6 animals, there was a consistent, yet non-significant delay in the pre-patent period, that is, the time to detection of blood stage parasites in the peripheral blood between wild-type and *ibis1^-^* knockout lines (Table S1). The *ibis1^-^* parasites did, however, display a significantly (*P* < 0.05) slower development of blood stage parasitemia in comparison with wild-type parasites after sporozoite injection (Fig. 2B). This slower parasite development was not detectable in animals infected with *IBIS1-mCherry* sporozoites (Fig. 2B, Table S1), supporting the notion that mCherry tagging does not affect the function of IBIS1 *in vivo* and targeting of the *IBIS1* locus does not lead to the parasite attenuation observed in the knockout line.

We next wanted to analyse whether the detected slow increase of blood stage parasitemia can be attributed to liver or blood stages of infection. Quantification of the parasite burden in the livers 40 h post infection revealed that *ibis1^-^* sporozoites transformed and developed normally in the livers of infected animals (Fig. 2C) and even caused a significant, but minor, increase in parasite burden. Hence, the deficiency is not caused by defects in liver stage maturation. In contrast, when infected red blood cells were transferred to naive recipient mice to analyse blood infection stages exclusively, *ibis1*^-^ parasites displayed slower growth compared with wild-type parasites (Fig. 2D), indicating that the reduced growth of the *ibis1*^-^ parasites after sporozoite infection is primarily due to impairment of blood stage development. This trend of reduced parasite growth in blood infection stages was also apparent in a second independent *ibis1* knock-out clone. We detected no delay in the ability of the *ibis1^-^* parasites to form schizonts *in vitro* (Fig. S5B), indicating that the defect is in egress, reinvasion, or related to clearance of infected red blood cells by the host animals.

### IBIS1 is exported to discrete structures in *P. berghei*-infected red blood cells

**Fig 3:**
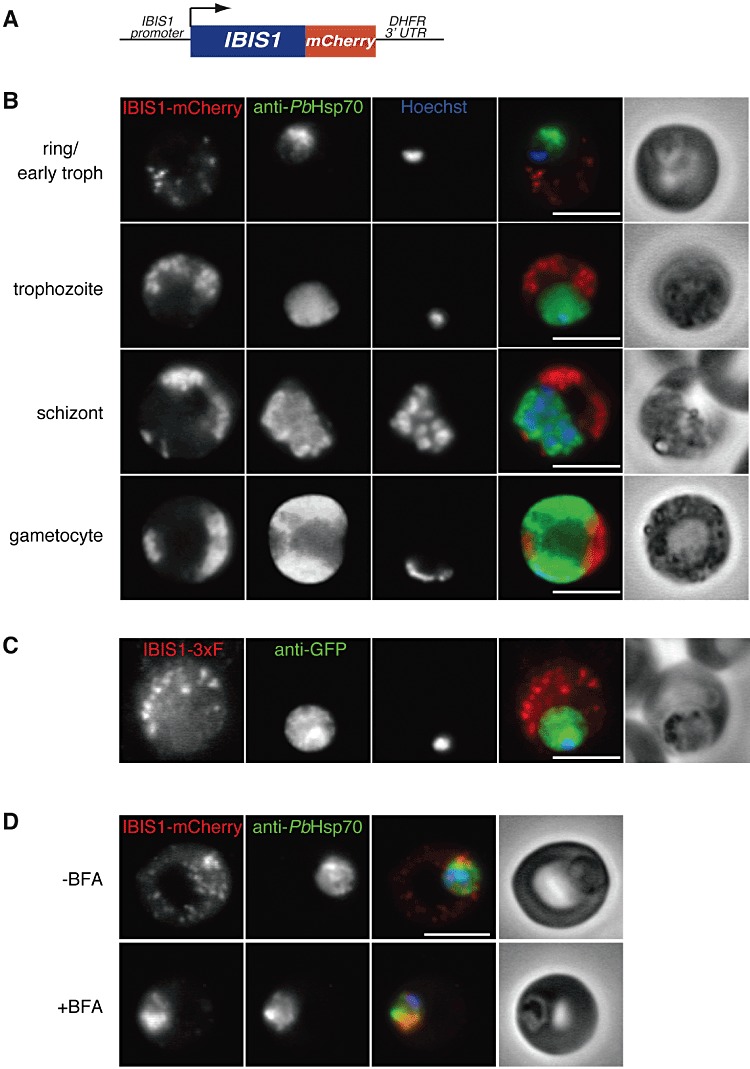
IBIS1 is expressed and exported from *P. berghei* throughout blood infection stages and localizes to discrete intra-erythrocytic structures. A. Schematic of the transgenic mCherry-tagged IBIS1 parasites (*IBIS1-mCherry*). The recombinant parasite line contains a carboxy-terminal fusion of the fluorescent mCherry tag (Shaner *et al*., 2004) obtained by insertional tagging of the endogenous *IBIS1* gene (see Fig. S2 for details). B. Mouse red blood cells infected with *IBIS1-mCherry* parasites were imaged. Cells were fixed and stained with anti-*Pb*HSP70 (centre left) along with Hoechst to visualize the parasites and parasite nuclei respectively. Phase-contrast images are shown on the right. C. IBIS1 tagged with 3xF has a similar localization in blood infection stages as compared with IBIS1-mCherry (B). Red blood cells infected with *IBIS1-3XF* parasites were fixed and stained with antibodies against FLAG and GFP to visualize IBIS1 and parasites respectively. Phase-contrast images are shown on the right. D. The export of IBIS1 depends on the parasite secretory pathway. Red blood cells harvested from a mouse infected with *IBIS1-mCherry* parasites were incubated for 3.5 h with BFA or DMSO as a control before fixation and visualization. Antibodies against *Pb*HSP70 (centre left) along with Hoechst (blue) to visualize the parasites and parasite nuclei respectively. Phase-contrast images are shown on the right. Scale bars, 5 µm.

Because our phenotypic assessment of the *ibis1*^-^ parasites indicates that *IBIS1* functions in blood stages of infection, we next investigated the localization of this protein in infected red blood cells using the *IBIS1-mCherry* parasite line (Fig. 3A). Consistent with these data as well as with microarray and proteome data (Hall *et al*., 2005), IBIS1 is detected throughout blood infection stages (Fig. 3B). In all stages the IBIS1-mCherry signal was detected exclusively in the infected red blood cell outside the parasite indicating export of IBIS1. Beginning in ring stage parasites, IBIS1-mCherry is localized to discrete punctate structures within the cytosol of the infected red blood cells. The IBIS1-mCherry signal increases over the course of parasite development, most likely because of continued protein synthesis. This localization to discrete puncta is independent of the fluorescent mCherry tag; IBIS1-3xF is also exported by *P. berghei* and displays a similar localization pattern (Fig. 3C). In gametocytes, we also detected IBIS1-mCherry (Fig. 3B) and IBIS1-3xF (data not shown) expressed and exported to the host erythrocyte cytosol.

To determine if IBIS1 export is dependent upon the parasite secretory pathway, as has been shown for several exported *P. falciparum* proteins (Crary and Haldar, 1992; Wickham *et al*., 2001; Przyborski *et al*., 2005) we inhibited transport from the *P. berghei* endoplasmic reticulum (ER) with the ARF guanine nucleotide exchange factor inhibitor Brefeldin A (BFA) (Fig. 3D). When red blood cells harvested from a mouse infected with *IBIS1-mCherry* parasites were incubated *in vitro* with BFA, the majority of the IBIS1-mCherry signal was retained inside the parasite, presumably within the parasite ER. In contrast, in control cells treated with DMSO, IBIS1-mCherry continued to be exported to the extra-parasitic punctate structures. We conclude that endogenous tagging of a novel PEXEL/HT-containing exported protein in the rodent malaria model parasite *P. berghei* delineates previously unrecognized punctate structures in mouse erythrocytes.

### IBIS1-delineated punctate erythrocyte structures are highly dynamic

**Fig 4:**
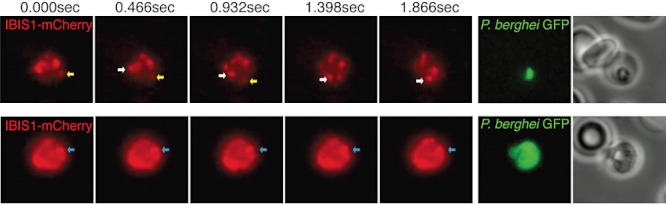
The intra-erythrocytic IBIS1-positive structures are highly motile during early stages of infection. Wide-field live imaging was used to visualize the IBIS1-mCherry signal of infected erythrocytes. The same field was imaged subsequently to visualize the GFP signal and phase image of the cells in addition to IBIS1-mCherry. Examples of an early trophozoite (top) and a late trophozoite (bottom) are shown. Yellow and white arrows indicated motile IBIS1-positive structures in the early trophozoite infected cell, and the blue arrow indicates a concentration of IBIS1, which remains immotile during the indicated time-course in the late trophozoite infected cell (bottom). The full movie including both cells is available online as Movie S1. Scale bars, 5 µm.

When the red blood cells infected with *IBIS1-mCherry* parasites were imaged live, the motility of these IBIS1-positive structures were immediately evident (Movie S1 and Fig. 4). In order to capture the fast movement of these structures in ring and trophozoite stages, we immobilized infected erythrocytes on Concanavalin A-coated glass dishes and imaged at approximately 5 frames per second. The IBIS1-positive structures appear to be less dynamic in later developmental stages. This decrease in motility could result from the reduced ability to distinguish individual structures caused by the increased IBIS1 signal; however, it would be consistent with the decreased mobility of Maurer's clefts observed in later stages of *P. falciparum*-infected red blood cells (Grüring *et al*., 2011).

### Membrane localization of IBIS1

**Fig 5:**
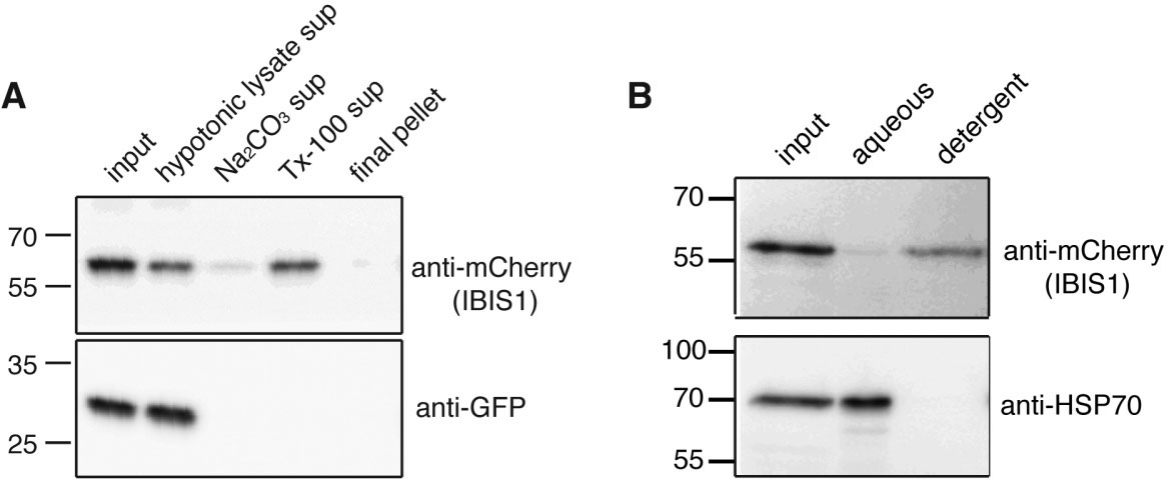
IBIS1 is a transmembrane protein A. Purified red blood cells infected with *IBIS1-mCherry* GFP ANKA parasites were lysed with hypotonic buffer (10 mM TRIS-HCl, pH 7.5) (input) and spun at 100 000 *g*. The supernatant (hypotonic lysate sup) along with proteins released from the pellet after either Na_2_CO_3_ treatment (Na_2_CO_3_ sup) or subsequent Tx-100 treatment (Tx-100 sup) and the remaining insoluble pellet were analysed by SDS-PAGE and Western blot. B. IBIS1 is an integral membrane protein. IBIS1-mCherry is primarily detected in the detergent fraction after purified infected red blood cells are subjected to Tx-114 extraction, whereas *Pb*HSP70 is detected exclusively in the aqueous fraction.

The primary structure of the IBIS1 protein contains a hydrophobic stretch predicted to function as a transmembrane domain in its carboxy-terminal portion (Fig. S1). We, therefore, wanted to investigate whether IBIS1 was in fact membrane-embedded during infection (Fig. 5). Initially, infected red blood cells were lysed hypotonically and lysates were spun at 100 000 *g* to pellet insoluble proteins and membranes (Fig. 5A). This pellet was treated with 1 mM Na_2_CO_3_ to release peripherally associated membrane protein and spun again at 100 000 *g*. The resulting pellet was solubilized in 1% Triton X-100 (Tx-100) and spun again. Approximately 50% of the IBIS1-mCherry protein was found in the supernatant after the first spin, and the remainder was mostly solubilized by Tx-100 (Fig. 5A). In contrast, the soluble control protein, GFP, was found exclusively in the supernatant following hypotonic lysis. Because half of the IBIS1-mCherry protein pelleted and could be released by Tx-100, we can conclude that at least this portion is present in membranes during infection. The proportion of the IBIS1-mCherry that is in the supernatant after hypotonic lysis could be a soluble trafficking intermediate, or alternatively, present in small vesicles that do not spin down efficiently at 100 000 *g*.

To confirm that IBIS1 is an integral membrane protein we tested the Triton X-114 (Tx-114) solubility of IBIS1 from *P. berghei*-infected mouse erythrocyte extracts. Because of its low cloud point, Tx-114 can be used to extract and separate membrane proteins in a lipid/detergent phase, while soluble and peripheral membrane proteins segregate to an aqueous phase (Bordier, 1981). When red blood cells infected with *IBIS1-mCherry* parasites were subjected to this phase separation, IBIS1 was detected in the lipid phase indicating that the protein is membrane-integrated during red blood cell infection (Fig. 5B). In contrast, the soluble control protein *Pb*HSP70 was detected exclusively in the aqueous fraction. These data together support the notion that the IBIS1-positive structures are membranous rather than proteinaceous in nature.

### Identification of small tubular membrane structures in *P. berghei*-infected erythrocytes

**Fig 6:**
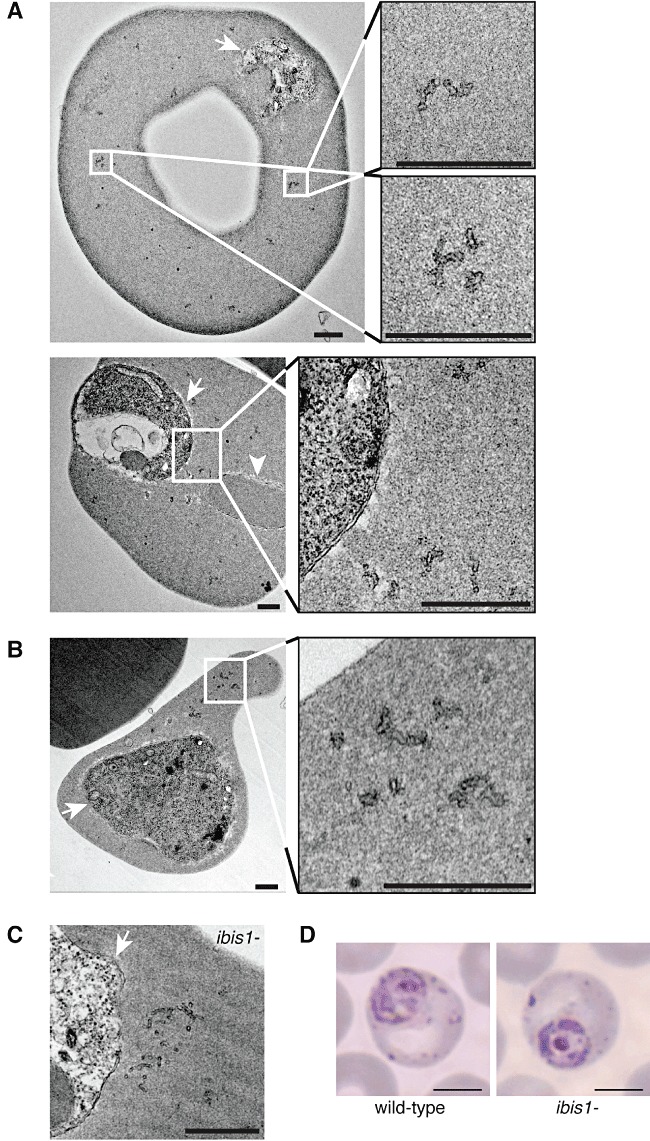
Transmission electron microscopy reveals membranous structures present in the red blood cell during *P. berghei* infection. A. Transmission electron micrographs of two representative red blood cells infected with early blood stage parasites (either ring or early trophozoite). The parasites are indicated with a white arrow. Several infected red blood cells contained the previously described large round membrane structures (arrowhead), and most infected cells contained multiple small tubular membrane structures (insets). B. Transmission electron micrographs of a red blood cell infected with a late trophozoite in which tubular membrane structures are again detectable in the red blood cell (insets). C. The membranous tubular structures also exist in the cytosol of blood cells infected with *ibis1*^-^ parasites. Transmission electron micrograph of a red blood cell infected with an *ibis1*^-^ parasite. The parasite is indicated with a white arrow. D. Giemsa-stained trophozoites from smears of peripheral blood 18 h after infection with mature schizonts. Note the presence of stained punctate structures throughout wild-type and *ibis1^-^* infected erythrocytes. Scale bars: 500 nm for electron micrographs, 3 µm for light micrographs.

The transmembrane nature of IBIS1 was our first indication that the IBIS1-positive puncta are membranous structures. Thus, we expect to find membranous structures in the cytosol of infected host cells throughout *P. berghei* development in red blood cells. The single published description of transmission electron microscopic analysis (TEM) of *P. berghei-*infected red blood cells reported two types of intra-erythrocytic structures, small vesicles at the cell surface and large circular membranous structures within the cytosol (Mackenstedt *et al*., 1989). Because of their apparent size, number and localization within infected cells, we suspect that neither of these described structures represents the structures we detect with the IBIS1 protein. We performed TEM on *P. berghei-*infected red blood cells harvested from infected mice to see if we could identify any additional membranous structures in the cytosoplasm of the host cells. We detected the previously described large circular membranes in infected cells. Interestingly, in the majority of infected cells we also detected small tubular membrane structures throughout the erythrocyte cytoplasm that had not been described previously. These structures were found in multiple infection stages (Fig. 6A and B). We could not detect these structures in uninfected red blood cells including reticulocytes. Reticulocytes contain several membranous structures as well as numerous ribosomes; however, the structures we observed in reticulocytes do not resemble the tubular structures we detect in the infected cells (Fig. S6). Nevertheless, we do not exclude the possibility that these tubular structures are derived from structures already present in the host cell prior to infection. The frequency of these tubular structures in infected cells, their absence from uninfected reticulocytes or erythrocytes and our biochemical data demonstrating the membrane nature of IBIS1 indicate that these structures could in fact be the IBIS1-positive structures we detect by fluorescence microscopy.

**Fig 7:**
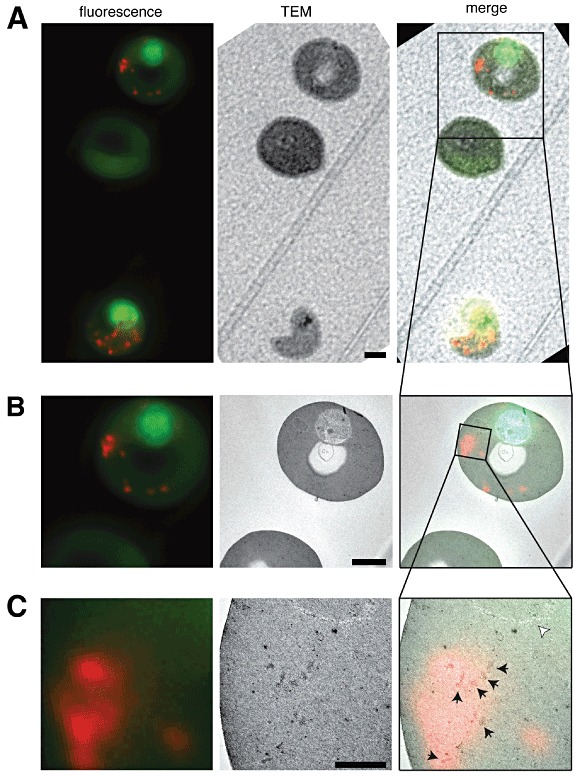
The location of tubular membrane structures visualized by TEM correspond to fluorescent IBIS1-mCherry-positive structures. Infected cells immobilized on ConA-coated, gridded coverglass surface were imaged by fluorescence microscopy (left panels). Cells were subsequently prepared for TEM, imaged (middle panels) and electron micrographs were correlated to the fluorescent images. Correlation with the fluorescent image was established with the lowest magnification TEM micrographs taken at 1100× (A), and subsequently aligned to those of taken at 4400× (B) and 50 000× (C). The tubular membrane structures of interest are marked with black arrows, and the previously described large circular membrane structure is indicated with a white arrowhead. Scale bars, 5 µm (A and B) or 500 nm (C).

In an attempt to confirm if these structures we detect by TEM are in fact the IBIS1-positive structures in infected red blood cells, we correlated TEM images with fluorescent images of the same cells. Prior to fixation and fluorescence imaging, infected red blood cells were immobilized on a gridded glass surface that enabled us to identify and orient the corresponding infected cells in the fluorescent images and on the EM grids. Indeed, the tubular membrane structures were found in regions of the blood cell in which IBIS1-mCherry signal was also detected (Fig. 7). Noticeably, tubular structures are not present for every fluorescent dot in the cell shown in Fig. 7; however, this is a predictable outcome because the entire depth of the red blood cell is visualized in the epifluorescence image of the cell whereas the electron micrograph shows only one plane of the cell. Still, we cannot rule out the possibility that IBIS1 localizes to a compartment distinct from these structures, nor can we exclude the possibility that these structures are not homogeneous and that IBIS1 localizes to only a subset of these tubular membranes. However, this correlation between the fluorescent and TEM images does provide evidence that these tubular membrane structures could be the IBIS1-positive structures seen by fluorescence microscopy.

Interestingly, no extra-parasitic structures have been yet described in Giemsa-stained *P. berghei*-infected red blood cells as is the case for *P. falciparum* and *P. vivax*. This prompted us to carefully re-examine Giemsa-stains of wild-type and *ibis1^-^* infected erythrocytes. In good agreement with our TEM data, we can detect some structures in trophozoite stage *P. berghei* stained with Giemsa, which could be the IBIS1-positive membranes (Fig. 6D). Of note, the absence of IBIS1 does not abolish these structures. This finding prompted us to perform TEM of *ibis1^-^* infected erythrocytes (Fig. 6C). Similar to the Giemsa-positive structures, we could also detect tubular structures by TEM, suggesting that the IBIS1-delineated structures may also be present in red blood cells infected with *ibis1^-^* parasites. These results indicate that IBIS1 is not required for the formation of these tubular structures; however, identification of additional proteins localizing to the IBIS1-positive structures is required to ultimately confirm a non-vital role of *IBIS1* in the formation of these structures.

### Expression of *P. falciparum* exported proteins in *P. berghei* reveals colocalization of Maurer's cleft proteins with IBIS1

To further characterize the compartment labelled by IBIS1 in infected erythrocytes, and to determine whether these IBIS1-positive structures correspond to known *P. falciparum* intra-erythrocytic structures, GFP-tagged *P. falciparum* proteins were introduced into *IBIS1-mCherry* parasites. All *P. falciparum* genes tested were flanked by the *IBIS1* 5′ and 3′ UTRs to control for similar expression and integrated into the *IBIS1* promoter (Fig. S7). Generation of these stable, recombinant, two-colour fluorescent parasites was achieved by selection for the human dihydrofolate reductase by daily treatment with the antifolate WR99210.

**Fig 8:**
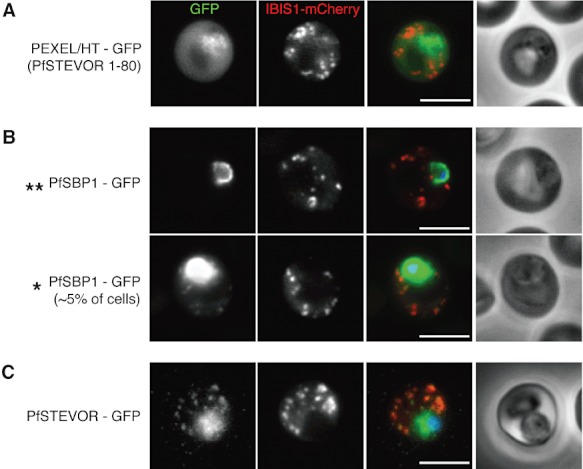
*P. falciparum* Maurer's cleft proteins colocalize with IBIS1 when expressed by *P. berghei*. Red blood cells from mice infected with *P. berghei* expressing both IBIS1-mCherry and the indicated C-terminally GFP-tagged *P. falciparum* proteins were fixed and cells that appeared to be infected with late ring or trophozoite stage parasites were imaged with epifluorescence microscopy. A. Coexpression of an amino-terminal fragment of *Pf*STEVOR (subtelomeric variable open reading frame; PFF1550w) containing the first 80 amino acid residues, including the PEXEL/HT motif, fused to GFP (Przyborski *et al*., 2005). Note the export of heterologous *Pf*STEVOR to the mouse erythrocyte and uniform distribution outside the parasite. B. Coexpression of GFP-tagged *Pf*SBP1 (skeleton binding protein 1), a signature protein of *P. falciparum* Maurer's clefts (Blisnick *et al*., 2000). Note that *Pf*SBP1-GFP remained associated with the parasite in the majority of infected red blood cells (marked **, top row), but in approximately 5% of cells, SBP1-GFP signal was detected in the red blood cell outside the parasite (marked *, bottom row). C. Coexpression of GFP-tagged full-length *Pf*STEVOR fused to GFP (Przyborski *et al*., 2005). Note substantial export of heterologous *Pf*STEVOR to the mouse erythrocyte and near-perfect colocalization with IBIS1-positive structures. Merge (centre right) and phase-contrast (right) are displayed for all panels. Scale bars, 5 µm.

As shown previously (MacKenzie *et al*., 2008), *P. berghei* is capable of exporting PEXEL/HT motif-containing proteins of *P. falciparum*, as evident by the translocation of the first 80 amino acids of *Pf*STEVOR (*Pf*STEVOR 1–80; PFF1550w) tagged with GFP out of the parasite into the host cytoplasm, resulting in a diffuse labelling of the erythrocyte cytoplasm (Fig. 8A).

One of the canonical markers of Maurer's clefts, *Pf*SBP1, is a so-termed PEXEL-negative exported protein (Blisnick *et al*., 2000). Heterologous expression in *P. berghei* revealed that *Pf*SBP1 is inefficiently exported (Fig. 8B). Intriguingly, in a small percentage of cells expressing *Pf*SBP1, low levels of exported *Pf*SBP1-GFP can be detected in puncta within the erythrocyte. In these cells, exported *Pf*SBP1 colocalizes with the IBIS1-positive structures.

This result prompted us to investigate the localization of full-length *Pf*STEVOR as an additional Maurer's cleft marker (Przyborski *et al*., 2005) in conjunction with IBIS1-mCherry. While some of the *P. berghei-*expressed *Pf*STEVOR was retained in the parasite, there was also significant *Pf*STEVOR-GFP signal colocalizing with the IBIS1 extra-parasitic puncta (Fig. 8C). Together, our data indicate that signature proteins of Maurer's clefts, an erythrocyte structure exclusively described in *P. falciparum*-infected erythrocytes, label IBIS in mouse erythrocytes infected with *P. berghei*.

## Discussion

Liver cells and red blood cells certainly present unique challenges to developing intracellular *Plasmodium* parasites. While liver cells have abundant lipids, amino acids and nucleotides to aid parasite development, they also possess intact innate host defences (Hafalla *et al*., 2011). Red blood cells, on the other hand, likely lack mechanisms to detect and combat intracellular pathogens; however, they are circulating and subject to clearance by the spleen and have finite resources for an intracellular pathogen to consume (Haldar *et al*., 2007; LeRoux *et al*., 2009). In both cell types, however, the parasites must evade these host defences and acquire nutrients for their development and replication.

Here we describe a *P. berghei* protein that is expressed and present at the host–parasite interface during both of these stages of infection. In infected liver cells, IBIS1 appears to be localized to the PVM and the TVN. At 16 h post infection, the TVN is extended and includes vesicles distributed quite distant from the parasite whereas in more mature liver stage parasites, IBIS1 is detected at the PVM just proximal to the parasite. In contrast, in infected red blood cells, IBIS1 is present at highly motile membranous structures throughout the red blood cell cytoplasm. Whether the apparent vesicles distal to the parasite in early infected liver cells derive exclusively from the PVM, as is suspected to be the case for Maurer's clefts in *P. falciparum*-infected red blood cells, remains to be studied. Also whether IBIS1 has the same function at these differing locations during the two infection stages remains to be determined.

Our data together with a recent study by Curra *et al*. (2012) show compartmentalization distinct from the parasitophorous vacuole in red blood cells infected with a rodent *Plasmodium* species. While we do not yet know the function of the newly described intra-erythrocytic *P. berghei*-induced structures, our initial findings suggest they could share some functions with Maurer's clefts in *P. falciparum*-infected red blood cells, although they are morphologically different and do not resemble flattened cisternae (Hanssen *et al*., 2010). Furthermore, they neither preferentially localize to the red blood cell periphery nor share morphological similarity to electron dense vesicles (EDV) (Hanssen *et al*., 2010). Our Tx-114 phase separation experiments demonstrate that IBIS1 is integrally associated with membranes in infected red blood cells, indicating that these structures are membranous. The detection of soluble IBIS1 after hypotonic lysis of infected red blood cells could indicate that IBIS1 is trafficked to these structures as a soluble intermediate as has been proposed for PfEMP1 and REX1 (Papakrivos *et al*., 2005; Dixon *et al*., 2008). In addition, the Maurer's cleft resident protein, *Pf*STEVOR localizes to the IBIS1-positive structures when expressed in *P. berghei*. Interestingly, the antibody against *P. falciparum* SBP1 has been shown to recognize a protein of approximately the correct size in *P. berghei* and *P. chabaudi* and also detects a non-homogeneous signal in the cytoplasm of red blood cells infected with these species, indicating that some of the described Maurer's cleft proteins may have un-annotated orthologues in *P. berghei* and other rodent malaria species (Blisnick *et al*., 2000).

One described role of Maurer's clefts is in the export of PEXEL/HT-containing proteins (Bhattacharjee *et al*., 2008). The *P. berghei* exportome consists of 37 proteins (Sargeant *et al*., 2006), and because these exportome predictions were based on known PEXEL/HT sequences from *P. falciparum*, this number is likely to be an underestimate. Previous work (MacKenzie *et al*., 2008) and our findings show that the mechanism of protein export in *P. berghei*-infected red blood cells is analogous to export from *P. falciparum*. It is therefore tempting to speculate that the extra-parasitic structures might be involved in PEXEL/HT-protein export in all *Plasmodium* species. Although still speculative, the IBIS1-positive structures in *P. berghei*-infected red blood cells are perhaps candidates to serve this function.

A well-established function of Maurer's clefts in *P. falciparum*-infected red blood cells is in the trafficking of parasite proteins to the red blood cell surface. Parasite proteins present on the surface of infected red blood cells are present transiently in Maurer's clefts prior to reaching the cell surface (Wickham *et al*., 2001; Wickert *et al*., 2003; Cooke *et al*., 2006). These surface proteins mediate the sequestration of the infected red blood cells to the vascular endothelium to prevent splenic clearance. While the molecular mechanisms of sequestration of *P. berghei*-infected red blood cells are not so well understood, it appears to be mediated by parasite proteins on the surface (Franke-Fayard *et al*., 2010; Fonager *et al*., 2012). If the function of IBIS1 contributes, either directly or indirectly, to protein transport to the red blood cell surface, perturbation of its function would be predicted to lead to increased clearance of infected red blood cells, which would in turn result in decreased parasitemia as seen for the *ibis1*^-^ parasites. However, we do not detect an increase in schizonts in the peripheral blood of *ibis1*^-^ infected mice (data not shown), as might be expected for a sequestration defect. Increased host clearance of infected red blood cells would perhaps account for why IBIS1 appears to contribute to efficient blood stages of infection but does not negatively influence parasite growth in liver infection stages: if IBIS1 has the same molecular activity in liver infection stages, the loss of IBIS1 function would not necessarily result in clearance of infected hepatocytes. While our *in vitro* data suggest that intracellular development of the parasites is not dramatically altered in red blood cells, an additional rational explanation for the phenotype we detect *in vivo* could be a decreased rate of *ibis1*^-^ parasite egress or reinvasion.

In conclusion, our findings of parasite-induced exomembrane structures in mouse erythrocytes infected with a rodent malaria parasite lend support to the hypothesis that host remodelling is far more conserved among *Plasmodium* species than previously anticipated.

## Experimental procedures

### Experimental animals, cell and parasite lines

Female NMRI and C57BL/6 mice were obtained from Charles River Laboratories. All animal work was conducted in accordance with the German ‘Tierschutzgesetz in der Fassung vom 18. Mai 2006 (BGBl. I S. 1207)’, which implements the Directive 86/609/EEC from the European Union and the European Convention for the protection of vertebrate animals used for experimental and other scientific purposes. The protocol was approved by the ethics committee of MPI-IB and the Berlin state authorities (LAGeSo Reg# G0469/09). Huh7 cells were cultured in supplemented RPMI. All parasite lines generated were either using *P. berghei* ANKA or *P. berghei* ANKA clone 507 parasites constitutively expressing green fluorescent protein (Janse *et al*., 2006). *Anopheles stephensi* mosquitoes were raised under a 14 h light/10 h dark cycle at 28°C and 80% humidity and were fed daily on 10% sucrose.

### Construction of plasmids

For C-terminal tagging of IBIS1, the C-terminal portion of *IBIS1* was amplified using the primers AI05 and AI06 cloned into the NotI and SpeI sites of the plasmid B3D+ mCherry (Silvie *et al*., 2008b). To tag IBIS1 with 3xF, the 3xF epitope tag was cloned into the SpeI and NotI sites of the vector b3D.DT^H.^D (provided by Dr Andrew Waters, Glasgow University) and the *IBIS1* coding region was amplified with primers AI160 and AI06 and the *IBIS1* 3′ UTR with primers AI161 and AI162 and were inserted into the vector between SacII and SpeI or NotI and BamHI respectively. To knockout *IBIS1*, the upstream and downstream genomic regions of *IBIS1* were amplified with the primers AI58, AI59, AI60 and AI61 and cloned into the SacII–NotI and HindIII–KpnI sites, respectively, of the B3D+ vector. Primers used in this study are summarized in Table S3.

### *P. berghei* transfections and genotypic analysis

For introduction of transgenes, C-terminal tagging and gene deletions, *P. berghei* parasites were transfected with digested plasmids using the Amaxa Nucleofector system as described (Janse *et al*., 2006). Transfected parasites were subsequently injected into naïve NMRI mice and selection was by oral pyrimethamine in the drinking water or injection of 0.3 mg WR99210 intraperitoneally with the previously described frequency (de Koning-Ward *et al*., 2000). Integration of the constructs was confirmed by PCR with specific primer pairs. To verify the *ibis1* knockout parasites, proper integration of the selection marker and removal of the *IBIS1* coding region was confirmed using the following primer pairs: (i) AI64 and AI65, which is within the coding region and should not amplify a product in *ibis1*^-^ parasites; (ii) AI62 and UTR, which reverse tests the 5′ integration site; and (iii) TgPro and AI63, which tests the 3′ integration site. The PCR products testing integration were subsequently sequenced to ensure that the integration occurred in the precise genomic region as designed.

### Infection of hepatoma cells *in vitro*

Huh7 cells were seeded in 8-chamber glass Lab-Tek slides (Nunc) and infected with 1.2 × 10^4^ sporozoites per well (Silvie *et al*., 2008b). Samples were subsequently fixed with 3% paraformaldehyde (PFA) in PBS and permeabilized with 0.3% Tx-100, and labelled with the following primary antibodies: anti-FLAG M2 (Sigma, 1:5000 dilution) polyclonal anti-GFP (Abcam, 1:1000 dilution), monoclonal *Pb*Hsp70 (Potocnjak *et al*., 1980), and poly-clonal rabbit anti-UIS4 (Müller *et al*., 2011) followed by Alexa 546- or Alexa 488-conjugated secondary antibodies (Invitrogen) and Hoechst 33342 (Invitrogen).

### Live imaging and immunofluorescence of infected red blood cells

Infected red blood cells were harvested from infected mice and immobilized on Concanavalin A (Sigma)-treated cover-slips or glass-bottomed dishes (Ibidi). For live imaging, cells were maintained in Ringer's solution (122.3 mM NaCl, 5.4 mM KCl, 1.2 mM CaCl_2_, 0.8 mM MgCl_2_, 11 mM d-glucose, 1 mM NaH_2_PO_4_ and 25 mM HEPES). Infected erythrocytes were recorded with wide-field microscopy. Cells on coverslips were fixed either with 4% PFA and 0.0075% glutaraldehyde in PBS as described (Tonkin *et al*., 2004) or with 4% PFA for 20 min are room temperature, followed by ice-cold methanol for 10 s. Antibody staining was performed with the reagents described above.

### Treatment of infected red blood cells with BFA

Infected red blood cells harvested from infected mice were cultured for 3 h 30 min in RPMI containing 20% FCS at 37°C in 5% CO_2_, 5% O_2_, 90% N_2_ with 5 µg ml^−1^ BFA or an equal volume of DMSO as a control. Subsequently, cells were immobilized on ConA-coated coverslips and prepare as described above for microscopy.

### *In vivo* phenotypic characterization of the *ibis1*^-^ line

Development of the parasites in the mosquito was performed by feeding mosquitoes on asynchronized blood stage parasites from infected NMRI mice. Exflagellation of gametocytes was checked prior to feeding, and wild-type and *ibis*^-^ parasites were fed in parallel to similarly aged mosquitoes. Sporozoites were dissected and counted after 17 days of development in the mosquitoes at 20°C.

To assess gametocyte production, mice were infected with 10^7^ infected red blood cells, and gametocyte production was assessed daily by comparing the number of gametocytes to the total number of parasites on smears of peripheral blood (at least 100 parasites per smear were counted). The day of highest gametocytemia is shown and occurred when the total peripheral blood parasitemia was between 3% and 5%.

The parasite burden in the liver after sporozoite infection was analysed as described (Friesen *et al*., 2010). Briefly, C57Bl/6 mice were injected with 10^4^ sporozoites intravenously. Forty hours post infection, livers of infected animals were harvested, homogenized, total RNA was extracted and relative parasite levels were determined with quantitative PCR by comparing the mean C*_t_* value of the *P. berghei 18 s ribosomal subunit* (Gene ID: 160641) to the mean C*_t_* value of the *Mus musculus gapdh* (Gene ID: 281199965) in the generated cDNA.

The further development of parasites in mice was followed by intravenous injection of either 1000 sporozoites dissected from infected mosquitoes or 1000 infected red blood cells isolated from mice with parasitemias between 0.3% and 1%. Parasitemia in the peripheral blood of the infected mice was subsequently measured daily by analysing Giemsa-stained blood smears. These growth experiments were repeated at least three times for the *ibis1*^-^ clone A line and two times for the *ibis1*^-^ clone B line (infected red blood cell infection only), and representative experiments are shown herein.

To assess schizont development *in vitro*, blood from infected mice in which the parasitemia was between 4.2% and 4.7% was used to inoculate cultures in RPMI containing 20% FCS and maintained at 36.4°C in 5% CO_2_, 5% O_2_, 90% N_2_. Samples were collected at the times indicated for smears that were subsequently stained with Giemsa. At least 150 parasites per slide were assessed. The experiment was repeated three times, and a representative experiment is shown.

### Biochemical fractionation

Infected red blood cells harvested from infected mice were purified on a Nycodenz gradient (Janse and Waters, 1995) and hypotonically lysed for 40 min on ice in 1 mM TRIS-HCl, pH 7.5. Lysates were spun at 100 000 *g* for 50 min. The pellet was resuspended in 1 mM Na_2_CO_3_ in PBS and spun again at 100 000 *g* for 50 min. The remaining pellet was resuspended in 1% Tx-100 in PBS, and spun again at 100 000 *g*. Equal amounts of each fraction were analysed by SDS-PAGE and Western blot using antibodies against mCherry and GFP (rat polyclonal, Chromotek, 1:1000 dilution).

For Tx-114 phase separation, Nycodenz-purified infected red blood cells were treated as described (Arnold and Linke, 2008; Pachlatko *et al*., 2010). Briefly, 1.5 × 10^7^ cells were lysed in 25 µl 0.09% saponin on ice followed by 225 µl Tx-114 buffer (1% Tx-114, 150 mM NaCl, 10 mM Tris-Cl pH 7.4). After incubation for 3 min at 30°C the samples were centrifuged at 300 *g*. The supernatant was removed and subject to a second round of Tx-114 extraction with 0.5% final concentration of Tx-114. After heating, centrifugation and separation, the aqueous phase was removed from the lower detergent phase and subject to a final rinse with 2% Tx-114 before the two phases were analysed by SDS-PAGE and Western blot analysis. Antibodies against mCherry (rat polyclonal, Chromotek, 1:1000 dilution) and *Pb*HSP70 (Potocnjak *et al*., 1980) followed by HRP-conjugated secondary antibodies were used to detect IBIS1-mCherry and *Pb*HSP70 in the samples.

### Electron microscopy

Infected red blood cells harvested from infected NMRI mice were immobilized on Concanavalin A-coated cover-slips or gridded Mattek dishes for correlation and fixed with 2.5% (v/v) glutaraldehyde and 2% (w/v) PFA in 100 mM cacodylate buffer (pH 7.4) for 30 min. Cells were rinsed three times for 5 min with 100 mM cacodylate buffer, postfixed for 1 h in 1% (v/v) osmium tetroxide, rinsed three times with distilled water, *en bloc* stained with 0.5% (w/v) uranyl acetate, dehydrated through a graded ethanol series and finally embedded using EMBed 812 (EMS). Cells were cut en face and 70–90 nm sections were collected. Sections were counterstained with 4% (w/v) uranyl acetate followed by lead citrate. All samples were imaged on a Zeiss EM 900 transmission electron microscope equipped with a wide-angle CCD camera (TRS-System, Moorenweis, Germany).

## Supporting information

Additional supporting information may be found in the online version of this article. Please note: Wiley-Blackwell are not responsible for the content or functionality of any supporting materials supplied by the authors. Any queries (other than missing material) should be directed to the corresponding author for the article.

## References

[b1] Arnold T, Linke D (2008). The use of detergents to purify membrane proteins. Curr Protoc Protein Sci.

[b2] Bano N, Romano JD, Jayabalasingham B, Coppens I (2007). Cellular interactions of *Plasmodium* liver stage with its host mammalian cell. Int J Parasitol.

[b3] Bhattacharjee S, van Ooij C, Balu B, Adams JH, Haldar K (2008). Maurer's clefts of *Plasmodium falciparum* are secretory organelles that concentrate virulence protein reporters for delivery to the host erythrocyte. Blood.

[b4] Blisnick T, Morales Betoulle ME, Barale JC, Uzureau P, Berry L, Desroses S (2000). Pfsbp1, a Maurer's cleft *Plasmodium falciparum* protein, is associated with the erythrocyte skeleton. Mol Biochem Parasitol.

[b5] Bordier C (1981). Phase separation of integral membrane proteins in Triton X-114 solution. J Biol Chem.

[b6] Cooke BM, Buckingham DW, Glenister FK, Fernandez KM, Bannister LH, Marti M (2006). A Maurer's cleft-associated protein is essential for expression of the major malaria virulence antigen on the surface of infected red blood cells. J Cell Biol.

[b7] Crary JL, Haldar K (1992). Brefeldin A inhibits protein secretion and parasite maturation in the ring stage of *Plasmodium falciparum*. Mol Biochem Parasitol.

[b8] Curra C, Pace T, Franke-Fayard BMD, Picci L, Bertuccini L, Ponzi M (2012). Erythrocyte remodeling in *Plasmodium berghei* infection: the contribution of SEP family members. Traffic.

[b10] Dixon MW, Hawthorne PL, Spielmann T, Anderson KL, Trenholme KR, Gardiner DL (2008). Targeting of the ring exported protein 1 to the Maurer's clefts is mediated by a two-phase process. Traffic.

[b11] Dixon MW, Kenny S, McMillan PJ, Hanssen E, Trenholme KR, Gardiner DL, Tilley L (2011). Genetic ablation of a Maurer's cleft protein prevents assembly of the *Plasmodium falciparum* virulence complex. Mol Microbiol.

[b12] Elmendorf HG, Haldar K (1994). *Plasmodium falciparum* exports the Golgi marker sphingomyelin synthase into a tubovesicular network in the cytoplasm of mature erythrocytes. J Cell Biol.

[b13] Fonager J, Pasini EM, Braks JAM, Klop O, Ramesar J, Remarque EJ (2012). Reduced CD36-dependent tissue sequestration of *Plasmodium*-infected erythrocytes is detrimental to malaria parasite growth *in vivo*. J Exp Med.

[b14] Franke-Fayard B, Janse CJ, Cunha-Rodrigues M, Ramesar J, Buscher P, Que I (2005). Murine malaria parasite sequestration: CD36 is the major receptor, but cerebral pathology is unlinked to sequestration. Proc Natl Acad Sci USA.

[b15] Franke-Fayard B, Fonager J, Braks A, Khan SM, Janse CJ (2010). Sequestration and tissue accumulation of human malaria parasites: can we learn anything from rodent models of malaria?. PLoS Pathog.

[b16] Friesen J, Silvie O, Putrianti ED, Hafalla JC, Matuschewski K, Borrmann S (2010). Natural immunization against malaria: causal prophylaxis with antibiotics. Sci Transl Med.

[b17] Ginsburg H, Krugliak M, Eidelman O, Cabantchik ZI (1983). New permeability pathways induced in membranes of *Plasmodium falciparum* infected erythrocytes. Mol Biochem Parasitol.

[b18] Grüring C, Heiber A, Kruse F, Ungefehr J, Gilberger TW, Spielmann T (2011). Development and host cell modifications of *Plasmodium falciparum* blood stages in four dimensions. Nat Commun.

[b19] Haeggstrom M, Von Euler A, Kironde F, Fernandez V, Wahlgren M (2007). Characterization of Maurer's clefts in *Plasmodium falciparum*-infected erythrocytes. Am J Trop Med Hyg.

[b20] Hafalla JC, Silvie O, Matuschewski K (2011). Cell biology and immunology of malaria. Immunol Rev.

[b21] Haldar K, Murphy SC, Milner DA, Taylor TE (2007). Malaria: mechanisms of erythrocytic infection and pathological correlates of severe disease. Annu Rev Pathol.

[b22] Hall N, Karras M, Raine JD, Carlton JM, Kooij TW, Berriman M (2005). A comprehensive survey of the *Plasmodium* life cycle by genomic, transcriptomic, and proteomic analyses. Science.

[b23] Hanssen E, Carlton P, Deed S, Klonis N, Sedat J, DeRisi J, Tilley L (2010). Whole cell imaging reveals novel modular features of the exomembrane system of the malaria parasite, *Plasmodium falciparum*. Int J Parasitol.

[b24] Hiller NL, Bhattacharjee S, van Ooij C, Liolios K, Harrison T, Lopez-Estrano C, Haldar K (2004). A host-targeting signal in virulence proteins reveals a secretome in malarial infection. Science.

[b25] Janse CJ, Waters AP (1995). *Plasmodium berghei*: the application of cultivation and purification techniques to molecular studies of malaria parasites. Parasitol Today.

[b26] Janse CJ, Franke-Fayard B, Mair GR, Ramesar J, Thiel C, Engelmann S (2006). High efficiency transfection of *Plasmodium berghei* facilitates novel selection procedures. Mol Biochem Parasitol.

[b9] de Koning-Ward TF, Fidock DA, Thathy V, Menard R, van Spaendonk RM, Waters AP, Janse CJ (2000). The selectable marker human dihydrofolate reductase enables sequential genetic manipulation of the *Plasmodium berghei* genome. Mol Biochem Parasitol.

[b27] Külzer S, Rug M, Brinkmann K, Cannon P, Cowman A, Lingelbach K (2010). Parasite-encoded Hsp40 proteins define novel mobile structures in the cytosol of the *P. falciparum*-infected erythrocyte. Cell Microbiol.

[b28] Lanzer M, Wickert H, Krohne G, Vincensini L, Braun Breton C (2006). Maurer's clefts: a novel multi-functional organelle in the cytoplasm of *Plasmodium falciparum*-infected erythrocytes. Int J Parasitol.

[b29] LeRoux M, Lakshmanan V, Daily JP (2009). *Plasmodium falciparum* biology: analysis of *in vitro* versus *in vivo* growth conditions. Trends Parasitol.

[b30] Mackenstedt U, Brockelman CR, Mehlhorn H, Raether W (1989). Comparative morphology of human and animal malaria parasites. I. Host-parasite interface. Parasitol Res.

[b31] MacKenzie JJ, Gomez ND, Bhattacharjee S, Mann S, Haldar K (2008). A *Plasmodium falciparum* host-targeting motif functions in export during blood stage infection of the rodent malarial parasite *Plasmodium berghei*. PLoS ONE.

[b32] Marti M, Good RT, Rug M, Knuepfer E, Cowman AF (2004). Targeting malaria virulence and remodeling proteins to the host erythrocyte. Science.

[b33] Maurer G (1902). Die malaria perniciosa. Zentralbl Bakteriol Parasitenkunde.

[b34] Mueller AK, Camargo N, Kaiser K, Andorfer C, Frevert U, Matuschewski K, Kappe SH (2005). *Plasmodium* liver stage developmental arrest by depletion of a protein at the parasite-host interface. Proc Natl Acad Sci USA.

[b35] Müller K, Matuschewski K, Silvie O (2011). The Puf-family RNA-binding protein Puf2 controls sporozoite conversion to liver stages in the malaria parasite. PLoS ONE.

[b36] Pachlatko E, Rusch S, Muller A, Hemphill A, Tilley L, Hanssen E, Beck HP (2010). MAHRP2, an exported protein of *Plasmodium falciparum*, is an essential component of Maurer's cleft tethers. Mol Microbiol.

[b37] Papakrivos J, Newbold CI, Lingelbach K (2005). A potential novel mechanism for the insertion of a membrane protein revealed by a biochemical analysis of the *Plasmodium falciparum* cytoadherence molecule PfEMP-1. Mol Microbiol.

[b38] Potocnjak P, Yoshida N, Nussenzweig RS, Nussenzweig V (1980). Monovalent fragments (Fab) of monoclonal antibodies to a sporozoite surface antigen (Pb44) protect mice against malarial infection. J Exp Med.

[b39] Przyborski JM, Miller SK, Pfahler JM, Henrich PP, Rohrbach P, Crabb BS, Lanzer M (2005). Trafficking of STEVOR to the Maurer's clefts in *Plasmodium falciparum*-infected erythrocytes. EMBO J.

[b40] Rodrigues CD, Hannus M, Prudencio M, Martin C, Goncalves LA, Portugal S (2008). Host scavenger receptor SR-BI plays a dual role in the establishment of malaria parasite liver infection. Cell Host Microbe.

[b41] Rowe JA, Claessens A, Corrigan RA, Arman M (2009). Adhesion of *Plasmodium falciparum*-infected erythrocytes to human cells: molecular mechanisms and therapeutic implications. Expert Rev Mol Med.

[b42] Sargeant TJ, Marti M, Caler E, Carlton JM, Simpson K, Speed TP, Cowman AF (2006). Lineage-specific expansion of proteins exported to erythrocytes in malaria parasites. Genome Biol.

[b43] Shaner NC, Campbell RE, Steinbach PA, Giepmans BN, Palmer AE, Tsien RY (2004). Improved monomeric red, orange and yellow fluorescent proteins derived from *Discosoma* sp. red fluorescent protein. Nat Biotechnol.

[b44] Silvie O, Mota MM, Matuschewski K, Prudencio M (2008a). Interactions of the malaria parasite and its mammalian host. Curr Opin Microbiol.

[b45] Silvie O, Goetz K, Matuschewski K (2008b). A sporozoite asparagine-rich protein controls initiation of *Plasmodium* liver stage development. PLoS Pathog.

[b46] Singh AP, Buscaglia CA, Wang Q, Levay A, Nussenzweig DR, Walker JR (2007). *Plasmodium* circumsporozoite protein promotes the development of the liver stages of the parasite. Cell.

[b47] Spielmann T, Fergusen DJ, Beck HP (2003). etramps, a new *Plasmodium falciparum* gene family coding for developmentally regulated and highly charged membrane proteins located at the parasite-host cell interface. Mol Biol Cell.

[b48] Tarun AS, Peng X, Dumpit RF, Ogata Y, Silva-Rivera H, Camargo N (2008). A combined transcriptome and proteome survey of malaria parasite liver stages. Proc Natl Acad Sci USA.

[b49] Tonkin CJ, van Dooren GG, Spurck TP, Struck NS, Good RT, Handman E (2004). Localization of organellar proteins in *Plasmodium falciparum* using a novel set of transfection vectors and a new immunofluorescence fixation method. Mol Biochem Parasitol.

[b50] Trager W, Rudzinska MA, Bradbury PC (1966). The fine structure of *Plasmodium falciparum* and its host erythrocytes in natural malarial infections in man. Bull World Health Organ.

[b51] Wickert H, Wissing F, Andrews KT, Stich A, Krohne G, Lanzer M (2003). Evidence for trafficking of PfEMP1 to the surface of *P. falciparum*-infected erythrocytes via a complex membrane network. Eur J Cell Biol.

[b52] Wickham ME, Rug M, Ralph SA, Klonis N, McFadden GI, Tilley L, Cowman AF (2001). Trafficking and assembly of the cytoadherence complex in *Plasmodium falciparum*-infected human erythrocytes. EMBO J.

