# Supplementary material for: The exported Plasmodium berghei protein IBIS1 delineates membranous structures in infected red blood cells
Source: Mol Microbiol. 2012 Feb 21;83(6):1229–43. doi: 10.1111/j.1365-2958.2012.08004.x (PMC3502748; doi:10.1111/j.1365-2958.2012.08004.x)
Supplement: Supplementary file 1 — Additional supporting information may be found in the online version of this article. Please note: Wiley-Blackwell are not responsible for the content or functionality of any supporting materials supplied by the authors. Any queries (other than missing material) should be directed to the corresponding author for the article. [file mmi0083-1229-sd1.pdf]

Supporting Information for:

The exported *Plasmodium berghei* protein IBIS1 delineates membranous structures in infected red blood cells.

Alyssa Ingmundson, Carolin Nahar, Volker Brinkmann, Maik J. Lehmann, and Kai Matuschewski

Contents:

- **Supplemental Movie 1**
- **Supplemental Figures 1 - 7**
- **Supplemental Tables 1 - 3**

Supplemental Movie 1: High speed imaging of red blood cells infected with *IBIS1-mCherry* parasites.
Blood cells from mice infected with *IBIS1-mCherry* asexual blood stages were immobilized and recorded by wide-field fluorescence microscopy. Time lapse is indicated (upper right).

Ingmundson *et al.*, Suppl. Figure 1

A.

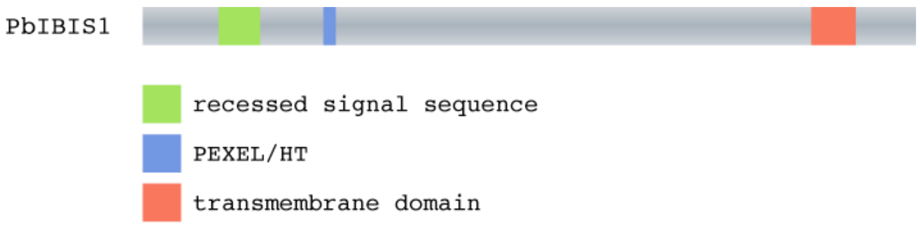

B.

```
           1                                                  50
PbIBIS1    MARNCECKKI DNDDMVSSTR YSNKFGEKFN LISCTKLFAL GMLFLICKNC
PyIBIS1    MARNFECKKI NSDDMTSSKK YSKNVGEKFN LISCTKLFAL SMLFLICQNY
PcIBIS1    ...MKKETKV DNGVIVPPTK HSQNWGKKLN LISCTKVFAL IVLFMICQNG

           51                                                100
PbIBIS1    DNSTQTTSAY QEYQYNGLIL GKSRILSELD QNKDQNLGYK TQSYEDSSTE
PyIBIS1    ENSPQSTSSH QEYQYNGLVL G.NRILSELD QAENHTISYK TNNYEDSSVE
PcIBIS1    DSSTQSTSSY QEYQHNGLVL GKSRILSELD KTETHATDYK TQSNEDSSVE

           101                                               150
PbIBIS1    NAST...... ..SYMSTLKT DEESNTAGED RKEDNNDAAF IGKKS.....
PyIBIS1    NPNQQT.... ..SDSSSLQT DE.......D KKKDDSDATS IGETSPTTET
PcIBIS1    DPNSPTIITE LASDSSKLKT DEESENEDTD KKKDDNDSTS IGETSSAGET

           151                                               200
PbIBIS1    .......... .......... .......... .......... ....TEKKPH
PyIBIS1    TSVEETVTIE DTESVEET.. ........ES VEETPSTSTE ETSSTGKKTY
PcIBIS1    TSAEEKLYTD IIDSILSSVV IDGKKIFEEK KESDGKNIFE KKPYDGKKLF

           201                                               250
PbIBIS1    IDRISSTLNP .......... .......... .......... .....ESTDR
PyIBIS1    VDRIASILNP LINGEKKSTE KK.......S SEKKSS.EKK SSDEQSSSDE
PcIBIS1    EEKKPSDEKK TPGEKKSSNE KKTPGEKKSS NEKKSSSEQK TSSEQKPSSE

           251                                               300
PbIBIS1    RKSSDEQKSS NDKNIFKDVD DLINGIKSRY QDITNKIKSP EFQNECKGYI
PyIBIS1    QNSSDDQNSF DDQKLFEDID NLINGIKSRY QEFSAKIKSP EFQNKCKSYM
PcIBIS1    QKPSSEQKPY DQKSFADDLD SIVNGIKSCY QEVANKVKSP EFQSACKEYV

           301                                               350
PbIBIS1    NVAKEMIEDH RNHAMRFVSR NLNSLGIDQI FNDE.HGGYA FLAKIMLTKV
PyIBIS1    NTAKEMIEER RNCAMSFISR NLNALGIDKI FEDE.FGGYA LLGKMMLTKV
PcIBIS1    NTAKELFDEH KSSALNFVSS SMQNLGINQI FDDENFGGFA TLGKLAVSKV

           351                                               400
PbIBIS1    FVDNLFVPNF LKNNSTILST IVYFLIISFI VSNYLDATQN NERERKNLNK
PyIBIS1    FIDNMFIPDF LRNSSTIILT IVYFLIMMFI VGSYLDINQD TKTERRNTNE
PcIBIS1    MVDNLFIPDY LKNQSPIILT VVYFLILFMT FGTIYDILPN NR.RRVNSNA

           401        411
PbIBIS1    SNIFCRAKPM .
PyIBIS1    SKLFNRTQPP M
PcIBIS1    PQTNYKPQPL .
```

Supplemental Figure 1: A schematic of the *P. berghei* IBIS1 protein (A), and alignment of the IBIS1 proteins from *P. berghei*, *P. yoelii*, and *P. chabaudi* (B) are colored to indicate the predicted signal sequence, PEXEL/HT motif, and transmembrane regions.

Ingmundson *et al.*, Suppl. Figure 2

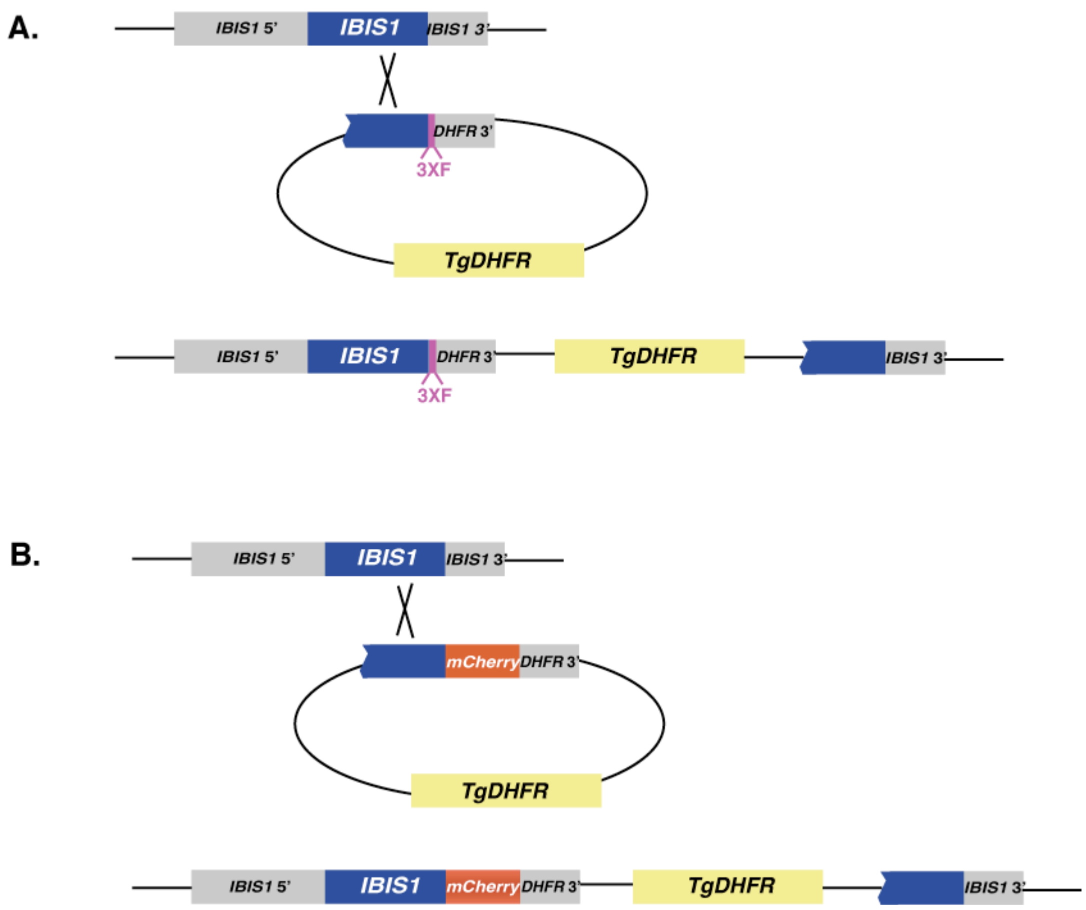

Supplemental Figure 2: Schematic for tagging *IBIS1*.
Endogenous *IBIS1* was tagged with (A) the triple FLAG (3xF) epitope or (B) the gene for the fluorescent protein mCherry by targeting vectors that result in tagged protein expressed from its endogenous promoter *via* single cross-over homologous recombination.

Ingmundson *et al.,* Suppl. Figure 3

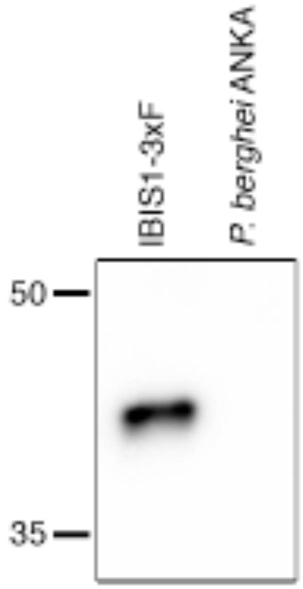

Supplemental Figure 3: Expression of IBIS1-3xF.
Protein extracts from mixed blood stage parasites isolated on a nycodenz gradient were probed with anti-FLAG antibody. IBIS1-3xF is detectable at the predicted size exclusively in *IBIS1-3xF* parasites and not in the wild-type parental isolate.

Ingmundson *et al.,* Suppl. Figure 4

A.

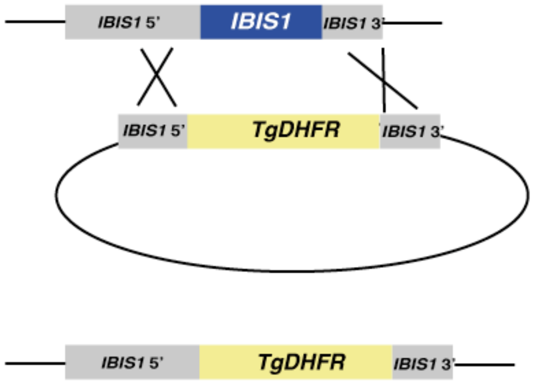

B.

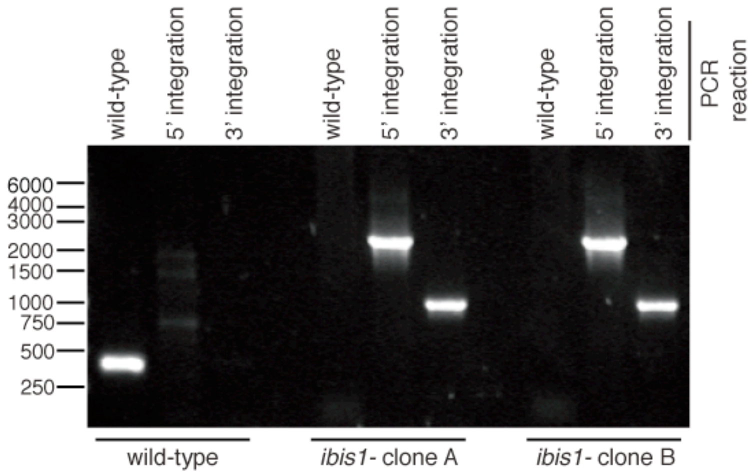

Supplemental Figure 4: Schematic for *IBIS1* gene deletion.
(A) *IBIS1* was deleted by replacement of the *IBIS1* coding region with a *TgDHFR* expression cassette.
(B) Correct integration of the plasmid was confirmed by PCR, and PCR products were sequenced to confirm correct integration of the selection marker and removal of *IBIS1*

Ingmundson *et al.,* Suppl. Figure 5

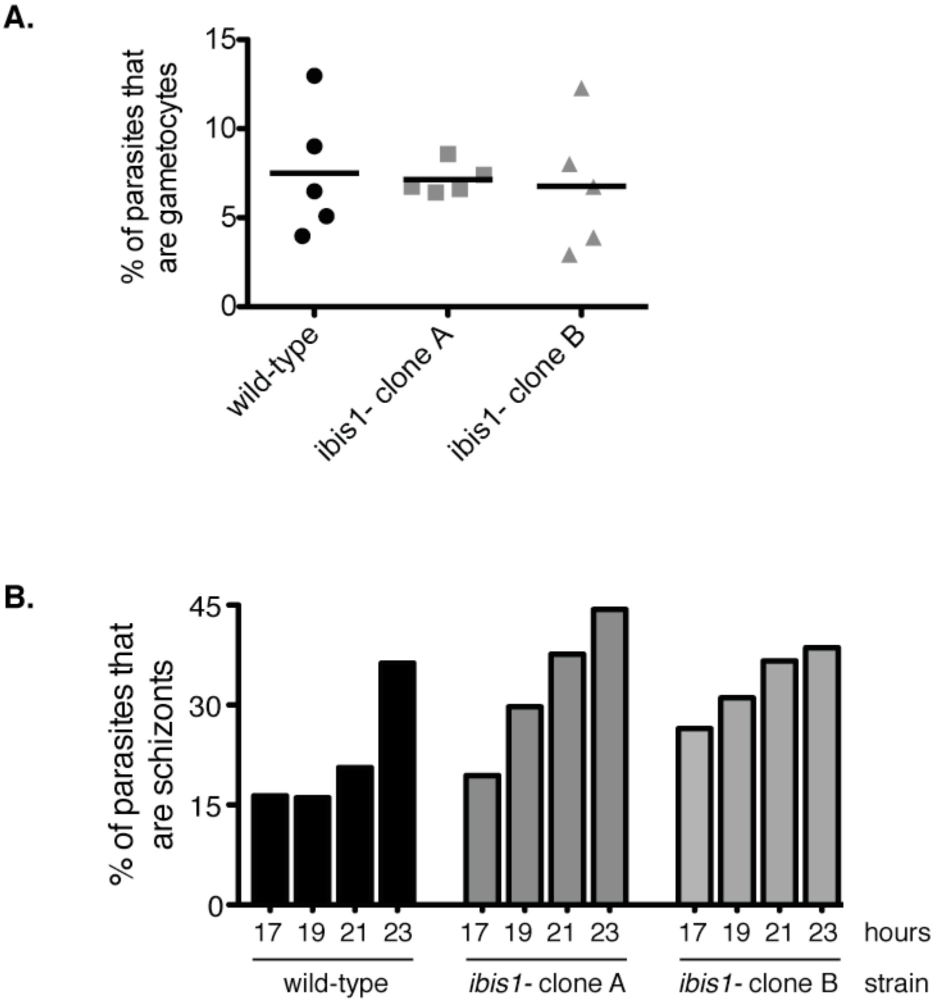

Supplemental Figure 5: *ibis1-* parasites display no defect in gametocytogenesis *in vivo* and no defect in schizont formation *in vitro.*

(A) Gametocytes were counted relative to the total number of parasites detected in smears of peripheral blood from mice infected with wild-type or *ibis1-* parasites. At least 100 parasites per mouse were assessed.

(B) Blood from infected mice was incubated in culture *in vitro* for 23 hours. Schizonts were counted relative to the total number of parasites detected in smears sampled from the cultures at the indicated times post-inoculation. At least 150 parasites per mouse were assessed. The experiment shown is representative of three experimental repetitions.

Ingmundson *et al.*, Suppl. Figure 6

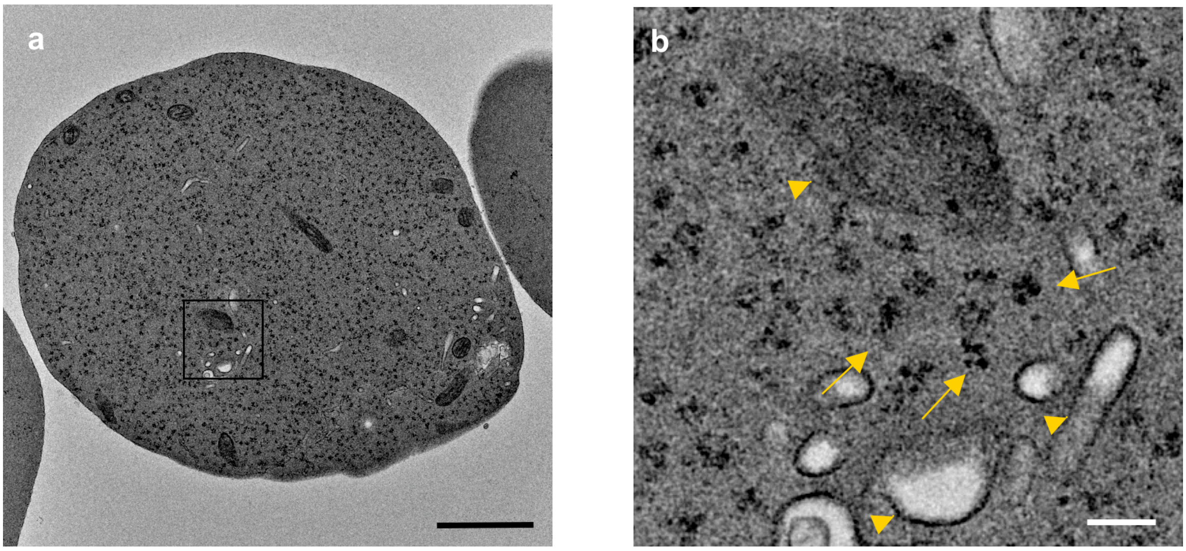

Supplemental Figure 6: Transmission electron micrographs of a representative reticulocyte. The tubular membranous structures found in *P. berghei* – infected red blood cells are not detected in reticulocytes. The electron dense structures indicated by yellow arrows are indicative of polyribosomes. Remnant organelles and some additional membranous structures present in these cells are marked by yellow arrowheads.
Scale bars: a: 1 mm , b: 100 nm

Ingmundson *et al.*, Suppl. Figure 7

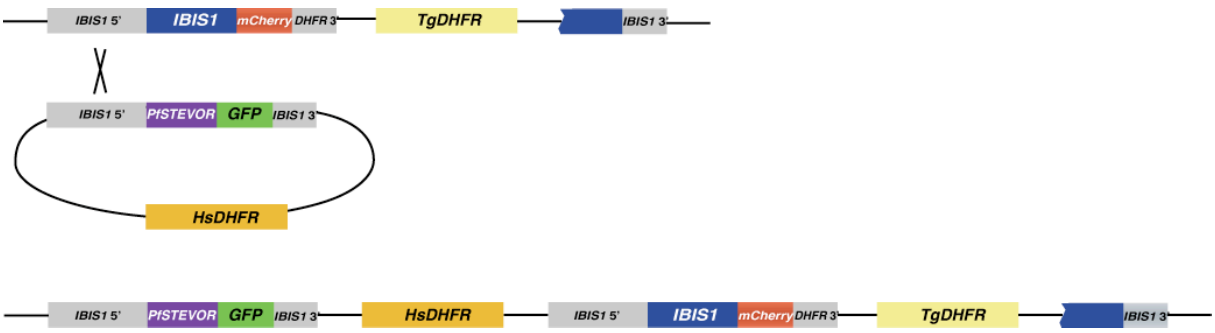

Supplemental Figure 7: Schematic to express GFP-tagged *P. falciparum* proteins in *P. berghei.*
GFP-tagged *P. falciparum* proteins were introduced under the control of the endogenous *IBIS1* promoter into the *IBIS1-mCherry* parasite line by targeting plasmids containing a second positive selectable marker (human dihydrofolate reductase; *HsDHFR*) permitting *in vivo* selection of recombinant parasite lines with the antifolate WR92110.

Supplemental table 1: Pre-patent period after injection of 1,000 sporozoites from 3 independent experiments.

	Average number of days to patency[a]			# of mice per group
Parasite line:	wild-type	*Ibis1-*	IBIS1-mCherry	
experiment 1	4.8	5	nt[b]	5
experiment 2	4.7	5.17	nt[b]	6
experiment 3	4.3	5.6	4.5	6

a. The time until the first parasite is detected in peripheral blood
b. Not tested

Supplemental Table 2: Summary of the *ibis1*- phenotype

Ibis1-line	Gameto-cytogenesis	Salivary gland sporozoites	Liver stage development	Blood stage development (following sporozoite infection)	Blood stage development (following infected-RBC infection)	*In vitro* schizont development
Clone A	WT	WT	+	–	–	WT
Clone B	WT	nt	nt	nt	–	WT

WT: equivalent to wild-type parental line
+ : greater parasite burden/numbers relative to wild-type parental line
– : decreased parasite burden/numbers relative to wild-type parental line
nt not tested

Supplemental Table 3: Primers used in this study.

AI05	atctgcggccgcCATCCGCATACCAAGAATACCAATATAATG
AI06	gatcactagtCATAGGTTTTGCTCTACAAAATATGTTAG
AI64	CTTATTTGTATTTTTACTTTTTAGAGCACGC
AI65	TTTGATGCCATTTATTAGATCATCAACATC
AI62	AAAATAATACATTTAATGAAGGGGAAAAGG
UTR rev	AATTCCGGTGTGAAATACCGCACAGATGCG
AI195	TTTAGTCGGCTCTGTTATAGTTTATATAAC
TgPro	CGCATTATATGAGTTCATTTTACACAATCC
AI160	tgcccgcggTGGCTCGaTAATTGCGAATGCAAAAAGATAG
AI161	taactacgcggccgcTGATTAAATTAATTATAAAAATAATATATGTATGCATATAC
AI162	acgggatccCATTAAAATATACAATTTCGATATAATATGTTGATC
AI58	caccgcggTCATACACTATACGTTTTTCCATTTGATGC
AI59	tctgtgcggccgcATATAATTAGATAACTAATTAATTATGTCC
AI60	acagaagcttCTTATATATATGTATGTGAGTTTGTACGCC
AI61	ctgtggtaccATACAACGAATCTCTGAACCTTTCTTATAC
